# Revisiting the HO^●^-initiated oxidation of L-proline amino acid in the aqueous phase: influence of transition metal ions

**DOI:** 10.1098/rsos.230114

**Published:** 2023-06-07

**Authors:** Dinh Hieu Truong, Thi Huong Lan Nguyen, Duy Quang Dao

**Affiliations:** ^1^ Institute of Research and Development, Duy Tan University, Da Nang 550000, Vietnam; ^2^ Faculty of Natural Sciences, Duy Tan University, Da Nang 550000, Vietnam; ^3^ Faculty of Pharmacy, Duy Tan University, Da Nang 550000, Vietnam

**Keywords:** DFT, l-proline, iron complexes, copper complexes, antioxidant, pro-oxidant

## Abstract

The oxidation of L-proline (Pro) by HO^●^ radical in water and the influence of transition metal ions on this process has been revisited by using the density functional theory (DFT) method at the M05-2X/6-311 + + G(3df,3pd)//M05-2X/6-311 + + G(d,p) level of theory at the temperature of 298.15 K. The main reactive sites of the HO^●^–initiated oxidation of Pro via hydrogen atom transfer (HAT) reactions are at the β- and γ-carbon, with the branching ratios being 44.6% and 39.5%, respectively. The overall rate constant at 298.15 K is 6.04 × 10^8^ M^−1^ s^−1^. In addition, Pro tends to form stable complexes with both Fe and Cu ions via the –COO functional group of dipole-salt form. The most stable Cu(II)-Pro complexes have high oxidant risks in enhancing the HO^●^ formation in the presence of reducing agents. Besides this, the high oxidation state metal complexes, i.e. Fe(III)-Pro and Cu(II)-Pro, may be oxidized by HO^●^ radical via HAT reactions but with a lower rate constant than that of free-Pro. By contrast, the low oxidation state metal complexes (i.e. Fe(II)-Pro and Cu(I)-Pro) have higher oxidation risks than the free ligands, and thus, the complexation enhances the oxidation of Pro amino acid.

## 1. Introduction

Amino acids exist in almost all natural species and play crucial biochemical roles for humans, animals, plants, fungi and microorganisms [1–5]. Among more than 500 natural amino acids discovered until 2020 [1], there are only 20 α-amino acids, plus selenocysteine, appearing in the genetic code [2]. In the biological environment, the amino acid consists of monomers for several biological macromolecular structures such as proteins, neurotransmitters, hormones and nucleic acids [3].

Several factors, such as free radicals and ultraviolet radiation, may oxidize the amino acids in different environments [6–14]. Hydroxyl radical (HO^●^) produced by the Fenton reagent, i.e. – Fe(II) + H_2_O_2_ system, consists of one of the most reactive oxidative factors investigated by Stadtman *et al.* [6]. The obtained products of the oxidation of amino acids by HO^●^ are ${\mathrm{NH}}_{4}^{+}$, α-ketoacids, CO_2_, oximes and aldehydes or carboxylic acids containing one less carbon atom. Furthermore, the relative oxidation rates of the studied amino acids varied as follows: L-leucine (Leu) > L-serine (Ser) > L-alanine (Ala) > glycine (Gly) > L-arginine (Arg) > L-tryptophan (Trp) > L-methionine (Met) > L-asparagine (Asp) > L-histidine (His). Besides, McGregor *et al.* [7] studied the reactions between singlet oxygen, O_2_ (^1^Δ_g_), with four amino acids, including His, Met, Trp and L-tyrosine (Tyr). As a result, the reaction rates varied from 5 × 10^5^ to 6 × 10^7^ M^−1^ s^−1^, with the decreasing order being: His > Trp > Tyr > Met. The influences of temperature and pH on the oxidation of Gly, Ala, Ser and L-threonine (Thr) in the aqueous phase by HO^●^ radical are experimentally and theoretically performed [8]. The rate constant increases from 1.3 to 3.0 times with a temperature varying from 278 to 318 K and from 1.2 to 3.6 times with a pH of 1.0 to 6.0.

Formation of complexes between amino acids and transition metal ions, such as Fe(III)/Fe(II) and Cu(II)/Cu(I), plays essential roles in many metabolisms in living creatures, i.e. electron transfer, catalysis, structural support and protein folding/unfolding [15–20]. However, these metal complexes may enhance the production of reactive HO^●^ radicals [21,22]. Milach *et al.* [21] found that L-glutamic acid (Glu), Ala, His and L-cysteine (Cys) and its derivatives favour the HO^●^ formation in the presence of cupric ion – Cu(II). Furthermore, Cys also enhances the HO^●^ production in the Fe(II)/EDTA system. Truong *et al.* [23] theoretically showed that Leu has high chelating potential toward Fe and Cu ions via its –COO functional group of dipole-salt form. The most stable Cu(II)-Leu complexes significantly promote the HO^●^ radical formation via Fenton reactions. Remarkably, the oxidant activity of complexes is enhanced considerably in the presence of an ascorbate anion.

**Figure 1.**
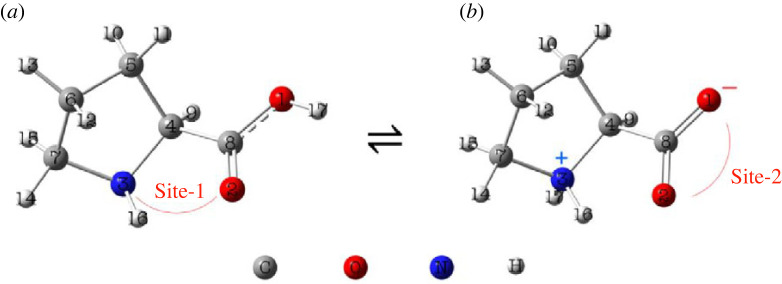
Optimized structures of L-proline in (*a*) neutral and (*b*) dipole-salt forms obtained in the aqueous phase at the M05-2X/6-311 + + G(d,p) level with numbered atoms and chelating sites.

L-(-)-Proline (Pro), a cyclic α-amino acid, is the only one of 20 DNA-amino acids having a secondary α-amine group (figure 1) [24]. Humans can produce it from arginine (Arg) and glutamine/glutamate (Gln) [24]. Pro is one of the main collagen components in the skin, tendons, bones and connective tissue, promoting their health and healing abilities. Pro plays a role as a biological antioxidant as reactive oxygen species (ROS) scavenger and singlet oxygen quencher [25,26]. Pro also prevents lipid peroxidation in alga cells exposed to heavy metals [27]. Direct oxidation of amino acid side chains of Pro, Arg, and L-lysine (Lys) may lead to the formation of glutamic semialdehyde and α-aminoadipicsemialdehyde, respectively, which are the protein carbonyl derivatives [28]. It is noted that high levels of protein carbonyls resulting from protein oxidation are implicated in either ageing [28,29] or the aetiology, progression or manifestations of several diseases such as rheumatoid arthritis [30], muscular dystrophy [31], and Alzheimer's disease [32]. Because of the direct relationship between protein oxidation and different diseases, several attempts in the literature have contributed to a better understanding of the Pro oxidation by reactive oxygen species (ROS) and other amino acids involved in the proteins. For example, Salamone *et al.* measured the rate constant for the HAT reaction between the cumyloxyl radical – [PheC(CH_3_)_2_]O^●^ with N-boc-protected Pro amino acid and its dipeptides [33]. The total rate constant of the reaction found in DMSO solvent at 25°C is (3.3 ± 0.2) × 10^6^ M^−1^ s^−1^. The authors suggested that the HAT reaction at the δ-C−H bond (i.e. C7 position, figure 1) is the predominant one due to the electron-withdrawing character of the α-substituents of the C−H bonds closer to the amino acid backbone. This polar effect deactivates the HAT reaction to electrophilic radicals such as HO^●^ or Cl^●^. Conversely, Signorelli *et al.* computationally indicated that the HO^●^-initiated oxidation of Pro occurs more favourably at the β- and γ-carbon positions (i.e. C5 and C6, figure 1) than those at the α- and δ-carbons (i.e. C4 and C7, figure 1) with lower reaction free energies of about 2 kcal mol^−1^ [34].

Although many works have focused on Pro's oxidation activity, its mechanism remains contrary and needs more attempts to shed light on the reaction kinetics and different influencing factors, such as temperature and transition metal ions. Thus, in this work, we revisit the HO^●^-initiated oxidation of Pro at the temperature of 298.15 K. For the first time, we investigate the effect of transition metal ions on the oxidation mechanism. First, we study the kinetics of the oxidation reaction by HO^●^ radical via formal hydrogen transfer (FHT) and single electron transfer (SET) mechanism. Second, the indirect effect of the complexation between Pro and Fe/Cu ions on the HO^●^ production is examined by evaluating the reduction reactions of the complexes towards two reducing agents, i.e. superoxide radical anion (O_2_^●–^) and ascorbate anion (Asc^–^), which are available in human body environment. Finally, the reactions between the most stable Fe-Pro and Cu-Pro complexes with HO^●^ radical are investigated to examine the direct effect in the oxidation process resulting from the metal ions' chelation.

## 2. Computational method

All geometry optimization and vibrational frequency calculations were performed by Gaussian 16 Rev. A.03 package [35]. All structures of reactants, transition states, pre-reactive complexes, post-reactive complexes and products were studied in the aqueous phase using M05-2X functional [36] and 6-311 + + G(d,p) basis set. Single-point calculations then improved the accuracy of the energy values at the M05-2X/6-311 + + G(3df,3pd) level of theory. The zero-point energy (ZPE) was corrected using a scaling factor of 0.961 [37,38]. The M05-2X functional has largely been chosen by several similar kinetic studies related to the complexes of transition metal and an organic ligand [23,39–41]. The influence of the aqueous media was mimicked using the solvation model based on the quantum mechanical charge density of a solute molecule interacting with a continuum description of the solvent (SMD) [42–44], which has also been widely used in the literature [39,41]. The structures of [Fe(H_2_O)_6_]^3+^, [Fe(H_2_O)_6_]^2+^, [Cu(H_2_O)_4_]^2+^ and [Cu(H_2_O)_4_]^+^ were employed as a recommendation by previous works [40,41,45–47]. The high-spin states of hydrated Fe(II) and Fe(III) ions in this study were equal to 5 and 6, respectively [23,41,46,48–51].

The kinetics of HO-initiated oxidation of Pro via FHT and SET reaction, the energetics for the chelation processes of Pro toward copper and iron ions, and the pro-oxidant risks of Pro determined via the reduction reactions of Fe(III)-to-Fe(II) and Cu(II)-to-Cu(I) complexes by two reducing agents, the superoxide radical anion (O_2_^●–^) and the ascorbate anion (Asc^−^) were resumed in the electronic supplementary material (SI file).

Finally, SEAGrid (www.seagrid.org) [52–55] is acknowledged for computational resources and services for the results presented in this publication.

## 3. Results and discussion

### 3.1. The oxidation of L-proline by HO^●^ radical

Optimized geometry for neutral and dipole salt forms of Pro in the aqueous phase are shown in figure 1. The frontier orbitals distribution and electrostatic potential maps are shown in electronic supplementary material, figure S1, and its Cartesian coordinates are shown in electronic supplementary material, table S1 (ESI). It is noted that the dipole-salt form is the primary form of Pro in the aqueous phase (approx. 100%). Thus, the overall rate constant of its oxidation reaction will be considered based on the dipole-salt form.

**Figure 2.**
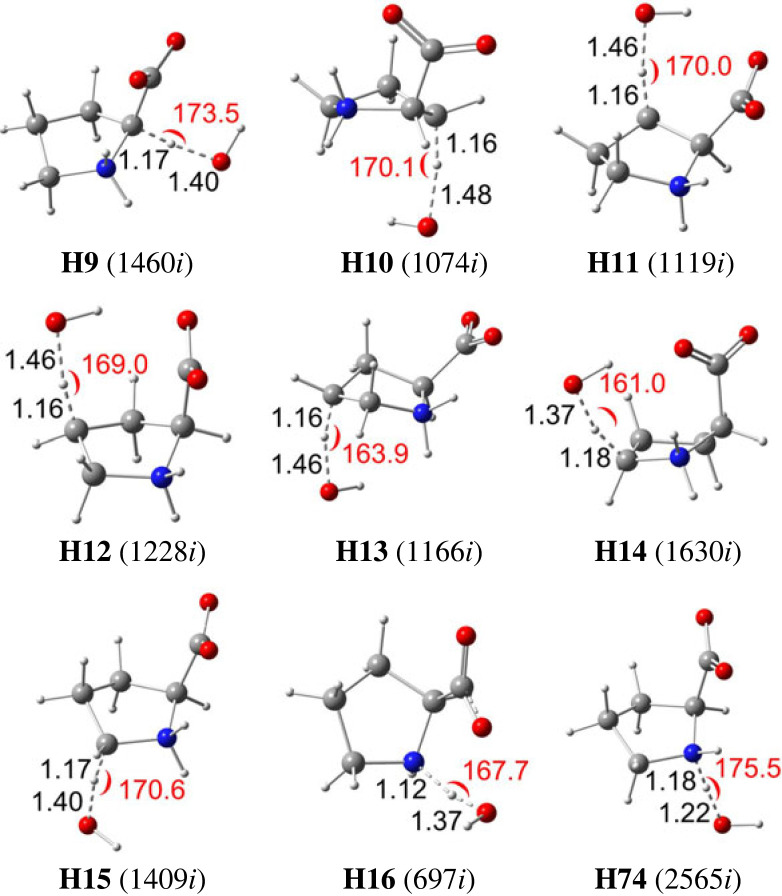
Optimized structures of the transition states (TSs) of FHT reactions between HO^●^ radical and the Pro-dipole-salt in water. Distances (in black colour) are in angstrom (Å), and angles (in red colour) are in degree (°). The numbers in parentheses are the imaginary frequency values.

In this section, the oxidation of Pro by HO^●^ radical is estimated by investigating the kinetics of the reactions occurring via two processes, formal hydrogen transfer (FHT) and single electron transfer (SET). Figure 2 demonstrates the optimized structures of the transition states (TSs) of the FHT reaction for the Pro-dipole-salt form occurring at all H atom positions. Similarly, the TSs structure of the Pro-neutral form is also calculated and presented in electronic supplementary material, figure S2 (ESI). The Cartesian coordinates and thermodynamic data of all the TSs are resumed in electronic supplementary material, table S2 (ESI file).

It is evident that the O···H distances between the transferring H atom and the O atom of HO^●^ radical vary from 1.22 to 1.48 Å, whereas the ones for C/N····H bonds at interactive sites range from 1.16 to 1.18 Å. Furthermore, the interactive angles of TSs, including ∠O··H··C and ∠O··H··N, vary from 161.0 to 175.5°.

The thermochemical and kinetics data FHT and SET reactions between the Pro-dipole-salt forms and HO^●^ radical in water at 298.15 K are presented in table 1. Meanwhile, those of Pro-neutral form are shown in electronic supplementary material, table S3 (ESI). It is noteworthy that the Δ_r_*G*^0^ of FHT reactions are negative, from −25.2 to −20.0 kcal mol^−1^; therefore, these reactions are, as expected, favourable and spontaneous. The most negative Δ_r_*G*^0^ value is found at the H9 position, being −25.2 kcal mol^−1^. By contrast, the Δ_r_*G*^0^ of SET reaction is highly positive, being 27.1 kcal mol^−1^. Regarding the Gibbs free energies of activation, the Δ*G*^‡^ values for FHT reactions of the dipole-salt form stand from 5.3 to 12.3 kcal mol^−1^. Conversely, the Δ*G*^‡^ of SET reaction is much higher, i.e. 86.5 kcal mol^−1^.

**Table 1.** The Gibbs free energies (Δ_r_*G*^0^, kcal mol^−1^), Gibbs free energies of activation (Δ*G*^‡^, kcal mol^−1^), diffusion rate constants (*k*_D_, M^−1^ s^−1^), thermal rate constants (*k*, M^−1^ s^−1^), apparent rate constants (*k*_app_, M^−1^ s^−1^) and branching ratios (*Γ*, %) of the FHT and SET reactions between the Pro-dipole-salt form with HO^●^ radical calculated in water at 298.15 K.

position	Δ_r_*G*^0^	Δ*G*^‡^	*k*_D_	*k*	*k*_app_	*Γ*
**FHT**						
H9	−25.2	7.7	3.03 × 10^9^	7.14 × 10^7^	6.98 × 10^7^	11.55
H10	−23.9	5.3	3.10 × 10^9^	1.93 × 10^8^	1.81 × 10^8^	30.04
H11	−23.9	6.5	3.08 × 10^9^	9.07 × 10^7^	8.81 × 10^7^	14.59
H12	−23.6	5.4	3.08 × 10^9^	1.63 × 10^8^	1.55 × 10^8^	25.60
H13	−23.6	5.7	3.08 × 10^9^	8.64 × 10^7^	8.41 × 10^7^	13.92
H14	−20.0	7.3	2.98 × 10^9^	1.52 × 10^7^	1.52 × 10^7^	2.51
H15	−20.0	7.3	3.03 × 10^9^	1.01 × 10^7^	1.01 × 10^7^	1.67
H16	−24.6	12.2	2.93 × 10^9^	5.03 × 10^2^	5.03 × 10^2^	0.00
H17	−24.6	12.3	2.83 × 10^9^	7.08 × 10^5^	7.08 × 10^5^	0.12
total FHT					6.04 × 10^8^	100.00
**SET**						
	27.1	86.5	8.09 × 10^9^	6.44 × 10^−50^	6.44 × 10^−50^	0.00
**Overall**						
*k*_overall_					**6.04 × 10^8^**	100.00

Furthermore, the diffusion-corrected apparent rate constants of FHT reactions for the dipole-salt form vary from 5.03 × 10^2^ to 1.81 × 10^8^ M^−1^ s^−1^, observed at the H16 (*Γ* 0.0%) and H10 sites (*Γ* 30.0%), respectively (figure 2). The reaction occurring at the H12 position also attracts our attention, with the rate constant as high as that at H10, i.e. 1.55 × 10^8^ M^−1^ s^−1^ (*Γ* 25.6%). The total branching ratios for the FHT reactions at H10/H11 (of C5 carbon) and H12/H13 (of C6 carbon), 44.6% and 39.5%, are remarkably higher than that at H9 (of C4 carbon, 11.5%) and H14/H15 (C7 carbon) (i.e. 4.2%). Our results agree with those computationally observed by Signorelli *et al*. [34], which indicated that the HO^●^-initiated oxidation of Pro in the aqueous phase occurs more favourably at the β- and γ-carbon positions than those at the α- and δ-carbon. Our observation is also in accordance with the ones found in previous studies. Galano *et al.* found that HO^●^ oxidizes Asp more favourably at the β-carbon (*k*, 2.66 × 10^9^ L mol^−1^ s^−1^) than at the α-carbon (*k*, 2.41 × 10^6^ L mol^−1^ s^−1^) at 298.15 K in the gas phase [56]. In the same reaction conditions, the authors observed that the HO^●^-initiated oxidation of Met via HAT reaction is more dominant at the γ-carbon (*k*, 1.66 × 10^10^ L mol^−1^ s^−1^) than at the α-carbon (*k*, 7.95 × 10^6^ L mol^−1^ s^−1^) [57]. Uranga *et al.* also confirmed that the first HO^●^ abstracts an H-atom from the β-carbon atom in the case of Ser and Thr, whereas γ-carbon and γ-sulfur atoms are the preferred sites for Met and Cys, respectively [12]. Alternatively, the SET reaction of the dipole-salt form of Pro is insignificant, with a negligible branching ratio.

In conclusion, the overall rate constant of the oxidation reactions is equal to 6.04 × 10^8^ M^−1^ s^−1^ at 298.15 K, which is close to the diffusion limit. This value is about 100 times higher than that obtained with N-boc-protected Pro oxidized by the cumyloxyl radical ([PhC(CH_3_)_2_]O^●^) in DMSO solvent at 25°C, with the value being (3.3 ± 0.2) × 10^6^ M^−1^ s^−1^ [33]. The obtained results can be explained by the fact that Pro exists in DMSO in both the neutral (30%) and dipole-salt forms (70%), which is quite different from our observation in water (approx. 100% dipole-salt form), and by the lower reactivity of the cumyloxyl radical compared to the hydroxyl one. Furthermore, this rate constant is significantly higher than the HO^●^-initiated oxidation of His, Met, Trp, Tyr and Ala [8] but lower than those of Asp [56], Ser [58] and Leu [59].

### 3.2. The complexation activity of L-proline towards transition metal ions

The complexation reactions of Pro were investigated with the ferric/ferrous and cupric/cuprous ions to shed light on the possibility of forming stable complexes between Pro and the transition metal ions. We evaluated the complexes of both neutral (Neu) and dipole-salt (DpS) forms. The neutral form of Pro has two chelating sites, including **O2-Neu**, and **Site-1**, while the dipole-salt has three chelating positions: **O1-DpS**, **O2-DpS** and **Site-2** (figure 1). For the mono-ligand complex, the chelation sites may be **O2-Neu**, **O1-DpS** and **O2-DpS** for monodentate complexes and **Site-1** and **Site-2** for bidentate complexes. The chelation sites for the di-ligand complexes having two Pro ligands are only found at **Site-2**. In this section, we only resume the results obtained with the dipole-salt form because of its predominance in the water.

#### 3.2.1. Iron ions chelation

**Figure 3.**
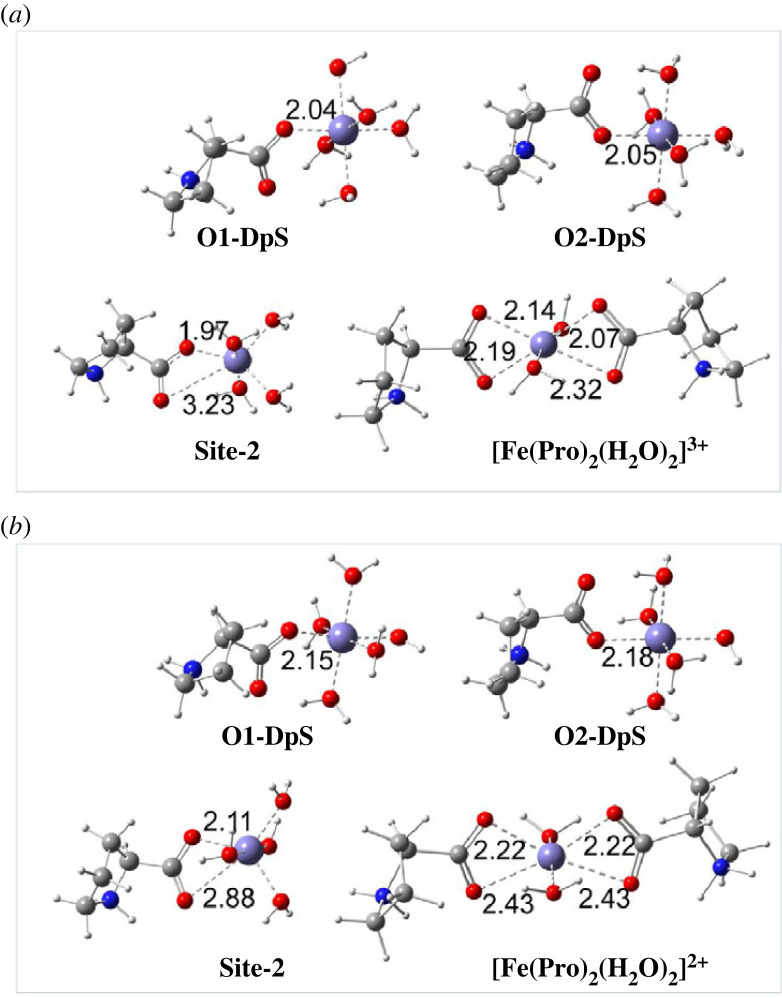
Optimized structures of four mono-ligand complexes at **O1-DpS**, **O2-DpS**, and **Site-2**, and one di-ligand complex at **Site-2** of Pro-dipole-salt with (*a*) Fe(III) ion and (*b*) Fe(II) ion in the aqueous phase. All distances are in Å.

Figure 3 displays the optimized structures for four mono-ligand and one di-ligand complexes types of the Pro-dipole-salt form with hydrated ferric [Fe(H_2_O)_6_]^3+^ and ferrous [Fe(H_2_O)_6_]^2+^ ions. It can be seen that the Fe–O distances vary from 2.04 to 2.05 Å for the Fe(III)-Pro monodentate complexes and from 1.97 to 3.23 Å for the bidentate ones. Meanwhile, the Fe–O lengths of the Fe(II)-Pro complexes are relatively more prolonged, varying from 2.15 to 2.18 Å and 2.11 to 2.88 Å, respectively. Cartesian coordinates and the thermochemistry data of all the Fe(III)/Fe(II)-Pro complexes and the hydrated Fe ions are displayed in electronic supplementary material, table S4 (ESI).

Gibbs free energies (Δ_r_*G*^0^) and formation constants (*K*_f_) obtained with the Pro-dipole-salt are resumed in table 2. The complete data of all Fe(III)/Fe(II)-Pro complexes are shown in electronic supplementary material, table S5 (ESI). As seen in table 2, the formation of all the Fe(III)-Pro and Fe(II)-Pro complexes in dipole-salt form is favourable and spontaneous, with the negative Δ_r_*G*^0^ values varying from −9.1 to −5.8 kcal mol^−1^ and −8.7 to −3.4 kcal mol^−1^, respectively. Their formation constant *K*_f_ is remarkably high, ranging from 1.71 × 10^4^ to 4.52 × 10^6^ and 3.33 × 10^2^ to 2.42 × 10^6^, in turn. **Site-2** is the preferable chelating site for both Fe(III)-Pro and Fe(II)-Pro complexes. The di-ligand complex at **Site-2** demonstrates the chelating ability of Pro at its higher concentration. We can see that the complexation possibilities toward Fe(III) and Fe(II) ions are likely to become more favourable and spontaneous, with the negative Δ_r_*G*^0^ being −15.5 and −14.3 kcal mol^−1^, respectively. The higher *K*_f_ are obtained, as 2.29 × 10^11^ and 2.96 × 10^10^, in turn, by doubling the concentration of Pro-dipole-salt.

**Table 2.** Gibbs free energies (Δ_r_*G*^0^, kcal mol^−1^) and formation constants (*K*_f_) of the complexation reactions between Pro-dipole-salt forms and Fe/Cu ions in the aqueous phase. Calculation are performed at M05-2X/6-311 + + G(3df,3pd)//M05-2X/6-311 + + G(d,p) level of theory.

	Fe(III)-Pro		Fe(II)-Pro		Cu(II)-Pro		Cu(I)-Pro	
	Δ_r_*G*^0^	K*_f_*	Δ_r_*G*^0^	K*_f_*	Δ_r_*G*^0^	K*_f_*	Δ_r_*G*^0^	K*_f_*
mono-ligand complex								
**O1-DpS**	−7.0	1.39 × 10^5^	−4.1	1.05 × 10^3^	−7.0	1.45 × 10^4^	−3.7	5.27 × 10^2^
**O2-DpS**	−5.8	1.71 × 10^4^	−3.4	3.33 × 10^2^	−6.8	9.34 × 10^4^	−3.5	3.42 × 10^2^
**Site-2**	−9.1	4.52 × 10^6^	−8.7	2.42 × 10^6^	−7.2	2.01 × 10^5^	−9.4	7.99 × 10^6^
di-ligand complex								
**Site-2**	−15.5	2.29 × 10^11^	−14.3	2.96 × 10^10^	−13.2	4.86 × 10^9^	−17.6	8.07 × 10^12^

Overall, the chelation abilities of Pro toward [Fe(H_2_O)_6_]^3+^ and [Fe(H_2_O)_6_]^2+^ ions are decided by the complexation of the dipole-salt form via its –COO functional group, including **O1**, **O2** and **Site-2** positions, to form stable complexes. The chelation ability of Pro becomes favourable in doubling the concentration of the ligand.

#### 3.2.2. Cu ions chelation

**Figure 4.**
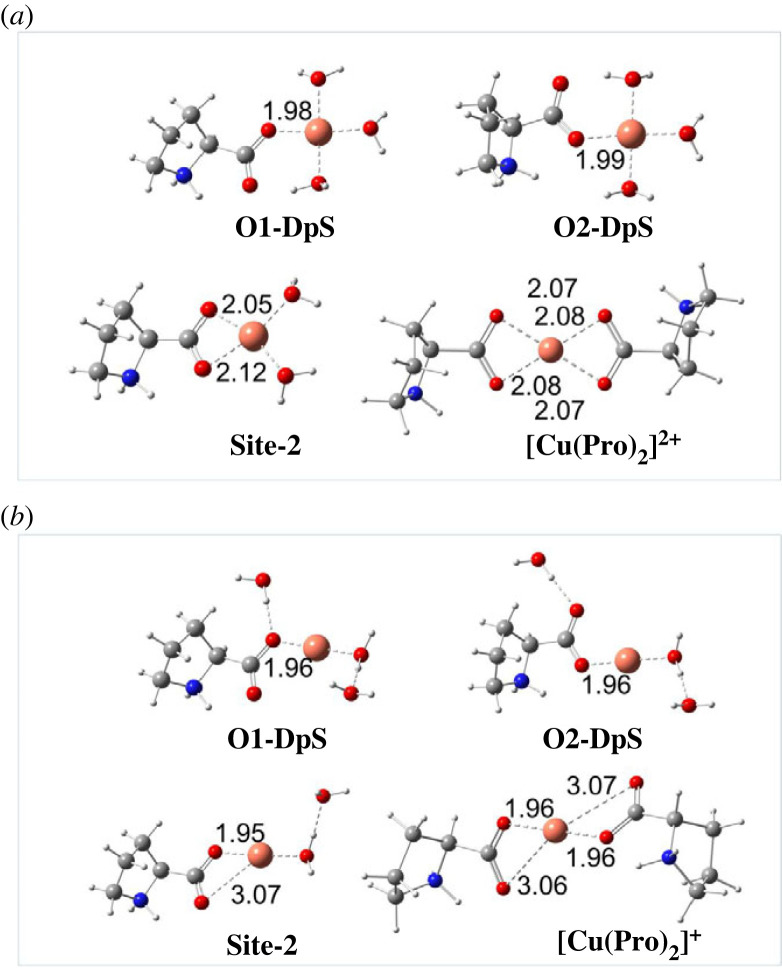
Optimized structures of four mono-ligand complexes at **O1-DpS**, **O2-DpS**, and **Site-2**, and one di-ligand Cu complex at **Site-2** of Pro-dipole-salt with (*a*) Cu(II) ions, and (*b*) Cu(I) ions in the aqueous phase. All distances are in Å.

The optimized structures of four mono-ligand and one di-ligand complexes types of dipole-salt Pro amino acid toward hydrated [Cu(H_2_O)_4_]^2+^ and [Cu(H_2_O)_4_]^+^ are shown in figure 4. Regarding Cu(II)-Pro complexes, Cu–O distances vary from 1.98 to 1.99 Å for monodentate and from 2.05 to 2.12 Å for bidentate complexes. The Cu–O lengths for Cu(I)-Pro complexes are about 1.96 Å and from 1.95 to 3.07 Å for mono- and bidentate complexes, respectively. Cartesian coordinates and the thermochemistry data of all the Cu-Pro complexes and the hydrated Cu ions are presented in electronic supplementary material, table S6 (ESI). Gibbs free energies (Δ_r_*G*^0^) and formation constants (K_f_) of all Cu(II)/Cu(I)-Pro complexes are also reported in electronic supplementary material, table S7 (ESI). The data obtained with the dipole-salt form is selectively given in table 2.

As seen in table 2, all the Cu(II)/Cu(I)-Pro complex formations of the dipole-salt form (DpS) are spontaneous and favourable. The Gibbs free energies of the stable complexes vary from −7.2 to −3.7 kcal mol^−1^ and −9.4 to −2.4 kcal mol^−1^ for Cu(II) and Cu(I) complexes, respectively. The corresponding formation constant K*_f_* values are from 5.02 × 10^2^ to 2.01 × 10^5^ and 5.43 × 10^1^ to 7.99 × 10^6^, in turn. Similar to the Fe complexes, **Site-2** is the most potentially chelating position toward Cu ions. With a double concentration of Pro, the chelation of Pro toward Cu(II) and Cu(I) at **Site-2** is significantly enhanced with Δ_r_*G*^0^ decreasing to −13.2 and −17.6 kcal mol^−1^, respectively, and the K*_f_* increasing to 4.86 × 10^9^ and 8.07 × 10^12^, in turn.

In conclusion, the Pro-dipole-salt form has a high potential to chelate [Cu(H_2_O)_4_]^2+^ and [Cu(H_2_O)_4_]^+^ via its oxygen atoms of the –COO functional group. The high concentration of Pro favours the complexations toward Cu ions via the **Site-2** position.

### 3.3. L-Proline oxidation enhanced by the transition metal ions

#### 3.3.1. Indirect enhancement

In this section, we try to address whether the complexation of Pro with transition metal ions, like Fe(III) and Cu(II), affects its oxidation. Indeed, several previous studies showed that some polyphenol-based molecules indirectly exhibit the reactive HO^●^ radical production via the Fenton-like reactions of their transition metal ion complexes [23,40,41,45–47]. The formed HO^●^ radicals have a high potential to return oxidizing Pro leading to the pro-oxidant risk of an organic molecule.

Thus, we evaluate the reduction reactions of Fe(III)-to-Fe(II) and Cu(II)-to-Cu(I) Pro complexes by reducing agents that can promote the initial step in the Fenton reactions, which later enhance the reactive HO^●^ radical production [41,46,60]. The reducing agents are superoxide anion ($O_{2}^{\;∙\text{–}}$) and ascorbate anion (Asc^–^), commonly in living bodies. The thermodynamic and kinetics data of these reactions are shown in electronic supplementary material, table S8 (ESI) for Fe complexes and electronic supplementary material, table S9 (ESI) for Cu complexes. The reactions of hydrated Fe(III)-to-Fe(II) and Cu(II)-to-Cu(I) ions are given as references. Herein, for a brief presentation, we only resume the corresponding data obtained for the Pro-dipole-salt in tables 3 and 4 for the Fe and Cu complexes, respectively.

**Table 3.** Gibbs free energies (Δ_r_*G*^0^), nuclear reorganization (*λ*), Gibbs free energies of activation (ΔG*^‡^*), the diffusion rate constants (*k*_D_), thermal rate constants (*k*) and the apparent rate constants (*k*_app_) of reduction reactions of Fe(III)-to-Fe(II) complexes by superoxide radical anion (O_2_^●–^) and ascorbate anion (Asc^–^) at 298.15 K. Units of energy values and rate constants are in kcal mol^−1^ and M^−1^ s^−1^, respectively.

position	Δ_r_*G*^0^	*λ*	Δ_r_*G*^‡^	*k_D_*	*k_TST_*	*k*_app_
${\left[\mathrm{Fe}{\left(H_{2}O\right)}_{6}\right]}^{3+}+O_{2}^{\;∙\text{–}}\;\to \;{\left[\mathrm{Fe}{\left(H_{2}O\right)}_{6}\right]}^{2+}+O_{2}$						
	−40.3	23.8	2.9	7.62 × 10^9^	4.84 × 10^10^	6.59 × 10^9^
${\left[\mathrm{Fe}(\mathrm{Pro}){\left(H_{2}O\right)}_{6-n}\right]}^{3+}+O_{2}^{\;∙\text{–}}\to {\left[\mathrm{Fe}(\mathrm{Pro}){\left(H_{2}O\right)}_{6-n}\right]}^{2+}+O_{2}$						
**O1-DpS**	−37.6	24.7	1.7	8.04 × 10^9^	3.78 × 10^11^	7.87 × 10^9^
**O2-DpS**	−38.1	24.2	2.0	8.04 × 10^9^	2.16 × 10^11^	7.75 × 10^9^
**Site-2**	−40.0	25.9	1.9	7.81 × 10^9^	2.39 × 10^11^	7.56 × 10^9^
${\left[\mathrm{Fe}{\left(\mathrm{Pro}\right)}_{2}{\left(H_{2}O\right)}_{2}\right]}^{3+}+O_{2}^{\;∙\text{–}}\to {\left[\mathrm{Fe}{\left(\mathrm{Pro}\right)}_{2}{\left(H_{2}O\right)}_{2}\right]}^{2+}+O_{2}$						
**Site-2**	−39.2	20.7	4.1	8.19 × 10^9^	6.12 × 10^9^	3.50 × 10^9^
[Fe(H_2_O)_6_]^3+^ + Asc^–^ → [Fe(H_2_O)_6_]^2+^ + Asc^●^						
	−14.5	26.5	1.4	7.44 × 10^9^	6.29 × 10^11^	7.35 × 10^9^
Fe(Pro)(H_2_O)_6−n_]^3+^ + Asc^–^ → Fe(Pro)(H_2_O)_6−n_]^2+^ + Asc^●^						
**O1-DpS**	−11.7	27.4	2.3	7.46 × 10^9^	1.39 × 10^11^	7.08 × 10^9^
**O2-DpS**	−12.2	26.9	2.0	7.46 × 10^9^	2.15 × 10^11^	7.21 × 10^9^
**Site-2**	−14.1	28.5	1.8	7.42 × 10^9^	2.89 × 10^11^	7.24 × 10^9^
[Fe(Pro)_2_(H_2_O)_2_]^3+^ + Asc^–^ → [Fe(Pro)_2_(H_2_O)_2_]^2+^ +Asc^●^						
**Site-2**	−13.3	26.5	1.1	7.50 × 10^9^	9.92 × 10^9^	7.44 × 10^9^

**Table 4.** Gibbs free energies (Δ_r_*G*^0^), nuclear reorganization (*λ*), Gibbs free energies of activation (ΔG*^‡^*), the diffusion rate constants (*k*_D_), thermal rate constants (*k*), and the apparent rate constants (*k*_app_) of reduction reactions of Cu(II)-to-Cu(I) complexes by superoxide radical anion ($O_{2}^{\;∙\text{–}}$) and ascorbate anion (Asc^–^) at 298.15 K. Units of energy values and reaction constants are in kcal mol^−1^ and M^−1^ s^−1^, respectively.

position	Δ_r_*G*^0^	*λ*	Δ_r_*G*^‡^	*k_D_*	*k_TST_*	*k*_ap*p*_
${\left[\mathrm{Cu}{\left(H_{2}O\right)}_{4}\right]}^{2+}+O_{2}^{\;∙\text{–}}\to {\left[\mathrm{Cu}{\left(H_{2}O\right)}_{4}\right]}^{+}+O_{2}$						
	−8.4	30.7	4.1	7.54 × 10^9^	6.64 × 10^9^	3.53 × 10^9^
${\left[\mathrm{Cu}(\mathrm{Pro}){\left(H_{2}O\right)}_{4-n}\right]}^{2+}+O_{2}^{\;∙\text{–}}\to {\left[\mathrm{Cu}(\mathrm{Pro}){\left(H_{2}O\right)}_{4-n}\right]}^{+}+O_{2}$						
**O1-DpS**	−5.2	31.6	5.5	7.87 × 10^9^	5.70 × 10^8^	5.32 × 10^8^
**O2-DpS**	−5.2	31.2	5.4	7.81 × 10^9^	6.85 × 10^8^	6.30 × 10^8^
**Site-2**	−10.5	30.4	3.3	7.84 × 10^9^	2.54 × 10^10^	5.59 × 10^9^
${\left[\mathrm{Cu}{\left(\mathrm{Pro}\right)}_{2}\right]}^{2+}+O_{2}^{\;∙\text{–}}\to {\left[\mathrm{Cu}{\left(\mathrm{Pro}\right)}_{2}\right]}^{+}+O_{2}$						
**Site-2**	−12.6	30.0	2.5	8.00 × 10^9^	8.97 × 10^10^	7.34 × 10^9^
[Cu(H_2_O)_4_]^2+^ + Asc^–^ → [Cu(H_2_O)_4_]^2+^ + Asc^●^						
	17.4	33.4	19.3	7.47 × 10^9^	4.11 × 10^−2^	4.11 × 10^−2^
[Cu(Pro)(H_2_O)_4−n_]^2+^ + Asc^–^ → [Cu(Pro)(H_2_O)_4−n_]^+^ + Asc^●^						
**O1-DpS**	20.6	34.3	22.0	7.43 × 10^9^	4.74 × 10^−4^	4.74 × 10^−4^
**O2-DpS**	20.6	33.9	21.9	7.42 × 10^9^	5.36 × 10^−4^	5.36 × 10^−4^
**Site-2**	15.3	33.1	17.7	7.42 × 10^9^	6.36 × 10^−1^	6.36 × 10^−1^
[Cu(Pro)_2_]^2+^ + Asc^–^ → [Cu(Pro)_2_]^+^ + Asc^●^						
**Site-2**	13.2	32.7	16.1	7.45 × 10^9^	9.65 × 10^0^	9.65 × 10^0^

Regarding the reactions of Fe(III)-to-Fe(II) complexes (table 3), the Fe complexes at the **Site-2** position for both mono- and di-ligand forms have similar or slightly higher Δ_r_*G*^0^ values (i.e. −40.0/−39.2 kcal mol^−1^ for $O_{2}^{\;∙\text{–}}$ and −14.1/−13.3 kcal mol^−1^ for Asc^–^ agent, respectively) than those of the hydrated ion [Fe(H_2_O)_6_]^3+^ (i.e. −40.3 and −14.5 kcal mol^−1^, respectively). The complexes at **O1-DpS** and **O2-DpS** also have higher Δ_r_*G*^0^ values than the [Fe(H_2_O)_6_]^3+^ ions. Thus, it is noteworthy that all the formed complexes of Fe(III) have a negligible risk of forming reactive HO^●^ radicals in the presence of $O_{2}^{\;∙\text{–}}$ and Asc^–^ reducing agents.

From the kinetics point of view, the results also show that the pro-oxidant risks resulting from the reduction reaction of Fe(III)-Pro complexes are negligible. The reactions of Fe(III)-to-Fe(II) mono-ligand complexes have the apparent rate constants *k*_app_ values from 7.56 × 10^9^ to 7.87 × 10^9^ M^−1^ s^−1^ for $O_{2}^{\;∙\text{–}}$ agent and 7.08 × 10^9^ to 7.24 × 10^9^ M^−1^ s^−1^ for Asc^–^, respectively. These rate constants are very close to those of hydrated Fe ions, i.e. 6.59 × 10^9^ and 7.35 × 10^9^ M^−1^ s^−1^, respectively (table 3). Similarly, the *k*_app_ of the reactions of Fe(III)-to-Fe(II) di-ligand complexes (i.e. 3.50 × 10^9^ and 7.44 × 10^9^ M^−1^ s^−1^) is similar to those of the hydrated Fe ions.

Regarding the Cu(II)-to-Cu(I) complexes reactions (table 4), the Δ_r_*G*^0^ values for **Site-2** mono- and di-ligand complexes towards $O_{2}^{\;∙\text{–}}$ reducing agents are all lower than one of the hydrated Cu ions (−10.5 and −12.6 kcal mol^−1^ versus −8.4 kcal mol^−1^). With the Asc^–^ agent, the Δ_r_*G*^0^ values for **Site-2** complexes are similarly lower than those of Cu ion. Meanwhile, the reduction reactions of mono-dentate complexes at **O1-DpS** and **O2-DpS** positions have higher Gibbs free energies than the ones of Cu ion reference.

Considering the corresponding rate constant, we can see that the mono-ligand Cu complexes at **Site-2** may induce pro-oxidant risks, with the rate constants being 5.59 × 10^9^ M^−1^ s^−1^ and 6.36 × 10^−1^ M^−1^ s^−1^ for the reaction with $O_{2}^{\;∙\text{–}}$ and Asc^–^, respectively. Meanwhile, the rate constants of the **Site-2** di-ligand complexes (i.e. 7.34 × 10^9^ and 96.5 × 10^−1^ M^−1^ s^−1^) are higher than those of hydrated Cu ions (i.e. 3.53 × 10^9^ and 4.11 × 10^−2^ M^−1^ s^−1^, respectively). Furthermore, the reaction rate constants increase when the number of Pro molecules increases from 1 to 2, from 6.36 × 10^−1^ to 96.5 × 10^−1^ M^−1^ s^−1^ for the reaction between the mono- and di-ligand Cu complexes with Asc^–^ agent. Thus, the bidentate complexes of the Pro-dipole-salt may produce HO^●^ radicals in the presence of the $O_{2}^{\;∙\text{–}}$ and Asc^–^ reducing agents.

In summary, the formation of the stable Cu(II)-Pro complexes may enhance the Fenton-like reaction when the reducing agents such as $O_{2}^{\;∙\text{–}}$ or Asc^–^ are available in the aqueous environment. In addition, this enhancement is remarkable when the concentration of Pro increases. However, the pro-oxidant risks of Fe(III)-Pro complexes are negligible.

#### 3.3.2. Direct enhancement

In this section, we investigate the oxidation of the formed Fe(III)- and Cu(II)-Pro complexes by HO^●^ radical and compare them with that of the free Pro. We will discuss the FHT reactions between the most stable mono-ligand bidentate **Site-2** complexes with HO^●^ radical at the two most reactive sites, i.e. H10 and H12, and SET ones in comparison with the corresponding reactions of the free Pro in the previous part.

**Figure 5.**
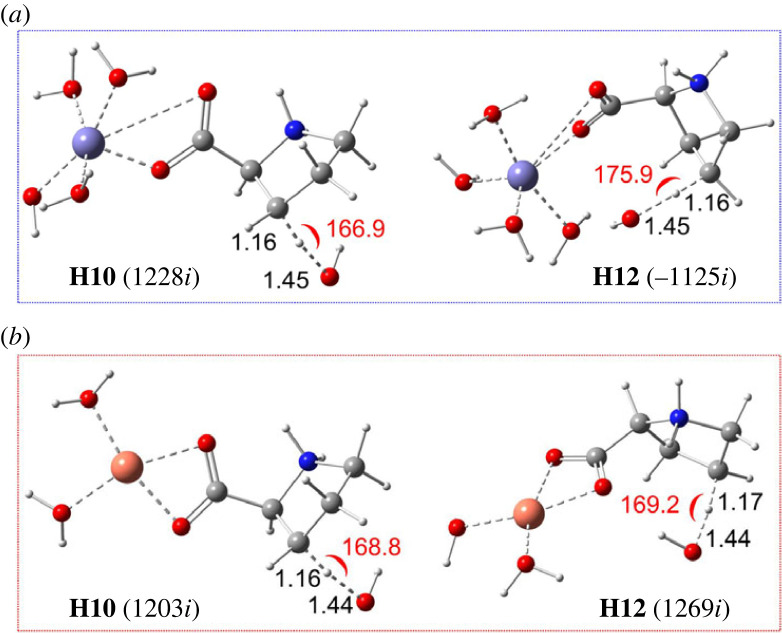
Optimized structures of the transition states (TSs) of FHT reactions between HO^●^ radical and Fe(III)-Pro complexes (*a*) and Cu(II)-Pro complexes (*b*) at H10 and H12 sites in water. Distances (in black colour) are in angstrom (Å), and angles (in red colour) are in degree (°). The numbers in parentheses are the imaginary frequency values.

**Figure 6.**
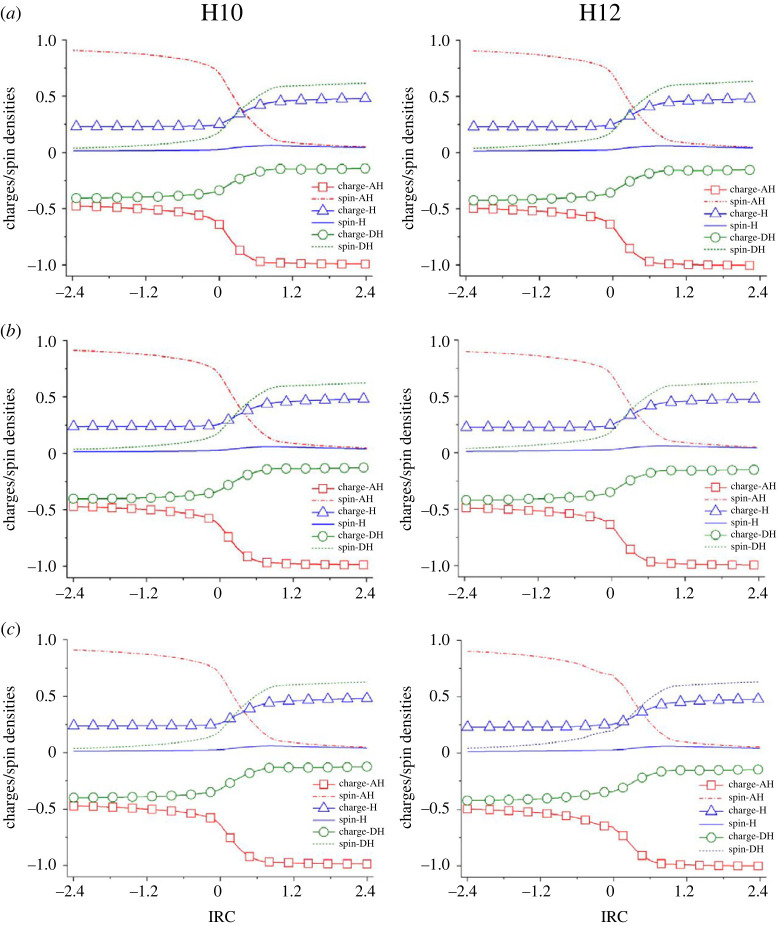
Evolution of NPA atomic charges and Hirshfeld spin densities for H-acceptor (AH), shifted-H (H), and H-donor (DH) as functions of the intrinsic reaction coordinate (IRC) between HO^●^ radical and dipole-salt form of Pro (*a***)**, Fe(III)-Pro complex (*b*) and Cu(II)-Pro complex (*c*) at H10 and H12 sites in the aqueous phase at the M05-2X/6-311 + + g(d,p) level.

Figure 5 represents the optimized structures for transition states of the FHT reaction between HO^●^ radical and the two **Site-2** complexes of Fe(III)-Pro and Cu(II)-Pro at H10 and H12. The Cartesian coordinates, and thermochemistry data of these structures are displayed in electronic supplementary material, table S9 (ESI). Figure 6 shows almost no difference between the TSs structures at the interactive sites for the complexes and the free ligand. Indeed, the C–H distances vary from 1.16 to 1.17 Å, while those of the O–H are from 1.44 to 1.45 Å, and the interactive angles vary from 166.9 to 175.9°, which are almost identical to those in the TSs of free Pro (figure 2). Despite many attempts, the TS structures for the Fe(II)-Pro and Cu(I)-Pro complexes cannot be located. As reported in previous studies, the FHT reactions between these complexes and HO^●^ radicals may likely transform into SET reactions with reaction rates close to the diffusion limit [61,62].

The kinetics of the reactions between Fe(III)-Pro and Cu(II)-Pro complexes with HO^●^ are resumed in table 5. As can be seen, the Gibbs free energies (Δ_r_*G*^0^) for the FHT reactions at the H10/H12 position are −23.6/−24.0kcal mol^−1^ and −23.3/−23.3 kcal mol^−1^ for Fe(III)-Pro and Cu(II)-Pro complexes, respectively. Furthermore, the rate constants are equal to 2.99 × 10^7^/7.10 × 10^6^ M^−1^ s^−1^ and 2.76 × 10^7^/1.84 × 10^7^ M^−1^ s^−1^, respectively. These values are similar to those of the free Pro (table 2). Similarly, the rate constants for SET reactions of the complexes are also as negligible as those of free Pro.

**Table 5.** The Gibbs free energies (Δ_r_*G*^0^, kcal mol^−1^), Gibbs free energies of activation (Δ*G*^‡^, kcal mol^−1^), diffusion rate constants (*k*_D_, M^−1^ s^−1^), thermal rate constants (*k*, M^−1^ s^−1^), apparent rate constants (*k*_app_, M^−1^ s^−1^) and branching ratios (*Γ*, %) of the FHT at H10 and H12, and SET reactions between the Fe and Cu complexes at **Site-2** with HO^●^ radical calculated in water at 298.15 K.

position	Δ_r_*G*^0^	Δ*G*^‡^	*k*_D_	*k*_TST_	*k*_app_
**[Fe Pro (H_2_O)_4_]^3+^**					
FHT-H10	−23.6	3.0	2.91 × 10^9^	3.02 × 10^7^	2.99 × 10^7^
FHT-H12	−24.0	2.9	2.93 × 10^9^	7.12 × 10^6^	7.10 × 10^6^
SET	48.5	83.5	8.18 × 10^9^	9.83 × 10^−48^	9.83 × 10^−48^
**[Fe Pro (H_2_O)_4_]^2+^**					
SET	−4.0	6.5	8.22 × 10^9^	2.69 × 10^9^	2.03 × 10^9^
**[Cu Pro (H_2_O)_2_]^2+^**					
FHT-H10	−23.3	2.2	2.89 × 10^9^	2.78 × 10^7^	2.76 × 10^7^
FHT-H12	−23.3	5.2	2.90 × 10^9^	1.85 × 10^7^	1.84 × 10^7^
SET	43.3	46.2	8.00 × 10^9^	2.19 × 10^−20^	2.19 × 10^−20^
**[Cu Pro (H_2_O)_2_]^+^**					
SET	−34.6	5.0	8.28 × 10^9^	3.48 × 10^10^	6.69 × 10^9^

As reported above, the reactions of Fe(II)- and Cu(I)-Pro complexes may occur via only the single electron transfer (SET) process, with the apparent rate constants close to the diffusion limit being 2.03 × 10^9^ and 6.69 × 10^9^ M^−1^ s^−1^ for Fe(II)-Pro and Cu(I)-Pro complexes, respectively. Those values are slightly higher than the overall rate constant of the free Pro, i.e. 6.04 × 10^8^ M^−1^ s^−1^ at 298.15 K (table 2). Overall, the complexation of Pro with the low oxidation state-based transition metal ions [Fe(II) and Cu(I)] enhances its oxidation rate by HO^●^ radicals more straightforwardly and more spontaneously via the SET reaction. Conversely, we do not observe any enhancement for the Pro oxidation when this amino acid chelates the high oxidation state of transition metal ions [i.e. Fe(III) and Cu(II)].

Finally, we attempt to provide deeper insight into the chemical nature of the FHT reaction between the free Pro amino acid towards HO^●^ radicals and the possible effect of the Fe(III) or Cu(II) ions complexation. We analyze in detail the NPA atomic charges and Hirshfeld spin densities for the H-acceptor (the O atom of HO^●^ radical), the transferred-H (H10 and H12), and the H-donor (the C atom at the interactive site) along the intrinsic reaction coordinates (IRC) of the reactions between free Pro, Fe(III)-Pro, and Cu(II)-Pro complexes and the HO^●^ at H10 and H12 sites. The singly occupied molecular orbitals (SOMO) and the SOMO-1 distributions for the transition states of the studied reactions are also evaluated.

### 3.4. Formal hydrogen transfer: HAT or PCET?

Hydrogen transfer, a critical step in many chemical, environmental and biological processes, is one of the fundamental chemical reactions. This process involves the transfer of two elementary particles, a proton and an electron, simultaneously or sequentially by hydrogen atom transfer (HAT) or proton-coupled electron transfer (PCET) process, respectively [63–66]. Distinguishing the chemical nature of these two processes has been a big challenge. In principle, HAT reactions which are adiabatic processes consist of the simultaneous transfer of an electron and proton from an H-donor to an H-acceptor without significant redistribution of molecular charge, corresponding to small solvent reorganization energies. Conversely, PCET reactions are non-adiabatic processes; thus, they typically involve different donors and acceptors for the electron and proton, and they are associated with significant molecular charge redistribution, tending to more powerful solvent reorganization energies. As a result, the electronic wave function of HAT reactions varies smoothly with proton coordination, whereas PCET reactions change suddenly and sharply [67]. Understanding these processes is crucial in evaluating critical biological reactions such as respiration, nitrogen fixation, photosynthesis and energy conversion in artificial photosynthesis or fuel cells [68]. Thus, in the final section of this paper, we contribute our attempts to answer whether the formal hydrogen transfer (FHT) between the free Pro amino acid with HO^●^ radicals occurs via HAT or PCET mechanisms, and the complexation towards the transition metal ions change the chemical nature of these mechanisms.

Figure 6 illustrates the evolution of NPA atomic charges and Hirshfeld spin densities for the H-acceptor (the oxygen atom of HO^●^ radical), shifted-H (H10 and H12) and H-donor (the carbon atom at the interactive site) along the intrinsic reaction coordinates (IRC) of the reactions between free Pro, Fe(III)-Pro and Cu(II)-Pro complexes with HO^●^ at H10 and H12 sites in the aqueous phase.

Regardless of the reaction of Pro at H10, the spin densities of the shifted-hydrogen (shifted-H) are relatively high, approximately 2.7 × 10^−2^ at the IRC point being 0.0 (TS point), and increase to values of about 6.2 × 10^−2^ at the product complex (PC) (figure 6*a*). These values correspond to hydrogen radicals transferred during a HAT reaction [64]. Furthermore, the spin density of the oxygen atom of HO^●^ radical (H-acceptor, AH) gradually decreases during the reaction path (0.91 at reactant complex – RC to 0.70 at TS and 0.05 at PC). The reverse trend of the carbon atoms (H-donor, DH) is observed in increasing from 0.04 (RC) to 0.19 (TS) and 0.62 (PC).

On the other hand, the NPA charges of the shifted-H are all small at the TS point, being approximately 0.25 e, corresponding to an H^●^ atom when forming a new O-H bond, and then significantly increase to the PC point (i.e. about 0.50 e). NPA charges of the oxygen atom (AH) remarkably decrease from −0.64 e (at RC) to −0.66 e (at TS) and −1.00 e (at PC) during the reaction due to the electron transfer from the shifted-H. Conversely, the charges of the carbon atom (DH) significantly increase from about −0.40 e (at RC) to −0.33 e (at TS) and −0.15 e (at PC). These observations are similar to the reactions at H10 and H12 and may correspond to the HAT reaction pathways in previous studies [64,65].

Regardless of the reaction of the Fe(III)-Pro and Cu(II)-Pro complexes (figure 6*b* and *c*), it is evident that the complexation toward Fe(III) and Cu(II) ions does not change the chemical nature of the oxidation mechanism with the similar observations for spin densities and atomic charges. Indeed, the spin densities of the shifted-H for the reactions for Fe(III)-Pro and Cu(II)-Pro complexes are about 2.7 × 10^−2^ and 2.8 × 10^−2^ and have the highest values at about 6.0 × 10^−2^ and 6.2 × 10^−2^, respectively. Moreover, the AH and DH spin densities for these reactions are close to those of free Pro. In addition, the NPA charges of shifted-H increase from about 0.24 e (at RC) to 0.26 e (at TS) and 0.48 e (at PC). The AH charges also decrease from −0.49 e (at RC) to −0.65 e (at TS) and −0.99 e (at PC). Meanwhile, the NPA charges of DH increase from −0.42 e (at RC) to −0.34 e (at TS) to −0.15 e (at PC). Overall, the complexation of Pro toward Fe(III) and Cu(II) ions does not change the chemical nature of HAT reactions between these complexes and HO^●^ radical.

**Figure 7.**
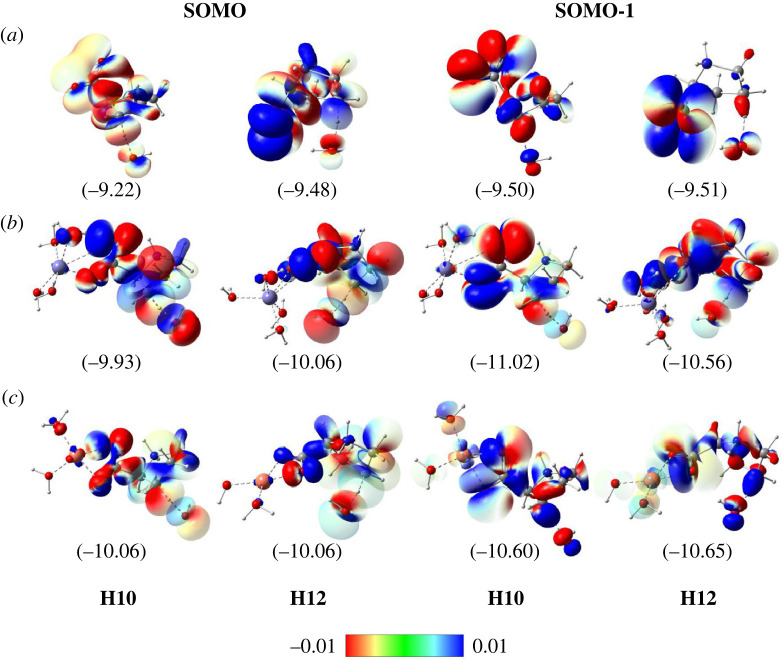
SOMO and SOMO-1 distributions at transition state geometries for the FHT reactions between (*a*) free Pro amino acid, (*b*) Fe(III)-Pro complexes and (*c*) Cu(II)-Pro complexes with HO^●^ radical at H10 and H12 sites in the aqueous phase calculated at the M05-2X/6-311 + + g(d,p) level (iso-value = 0.02). Values in parentheses correspond to orbital energies (Unit in eV).

Finally, the singly occupied molecular orbitals (SOMO) and SOMO-1 distributions for the transition states are evaluated and displayed in figure 7. The SOMO orbitals at the interactive sites lie on the AH····H···DH vectors and displays node plane at the shifted-H position, which has the SOMO orbital characteristics of the HAT transition state [63–65]. Moreover, for the free Pro and Cu(II)-Pro oxidation, the hydro transfer happens without the participation of the HOMO orbital (figure 7*a* and *c*). However, the Fe(III)-Pro and HO^●^ radical reaction is contributed by both SOMO and SOMO-1 orbitals. In addition, the SOMO and SOMO-1 energies vary from −10.06 to −9.93 eV and −11.02 to −10.56 eV, respectively, which are relatively lower than free Pro, from −9.48 to −9.22 eV and −9.51 to −9.50 eV, in turn. This observation explains why the oxidations of these complexes by HO^●^ are more difficult than free Pro, which is in good agreement with the kinetic results (table 5).

In summary, the oxidations of Pro and its complexes by HO^●^ radical occur via the HAT mechanism. In addition, the HAT reactions between Pro by HO^●^ become more complicated when Pro chelates transition metal ions, including Fe(III) and Cu(II).

## 4. Conclusion

The oxidation of L-proline (Pro) by HO^●^ radicals, as well as the influence of its complexation with Fe and Cu ions on this process, have been studied in the aqueous phase using the density functional theory (DFT) approach. The conclusions are multiple as follows:

— FHT reactions of dipole-salt form play a crucial role in the oxidation of Pro by HO^●^ radical, which occurs at various sites with the highest branching ratio found at H10 (30.04%) and H12 (25.60%). The total branching ratios for FHT reactions at C5 and C6 carbons are more predominant than those at C4 and C7. The overall rate constant is 6.04 × 10^8^ M^−1^ s^−1^ at 298.15 K. The obtained results agree with different works in the literature.
— Pro can spontaneously and favourably trap Fe and Cu ions via its –COO functional group of dipole-salt form, forming mono- and bi-dentate complexes. Furthermore, the chelating abilities increase by doubling its concentration.
— The indirect effect of transition metal ions complexation on the oxidation of Pro is complicated depending on the chemical nature of the metal ions. The Fe(III)-Pro complexes do not induce any remarkable risk of reactive HO^●^ radical formation. By contrast, the complexation of Pro toward Cu(II) ions enhances the appearance of the HO^●^ radicals via the Cu(II)-to-Cu(I) Pro complex formation in the presence of $O_{2}^{\;∙\text{–}}$ and Asc^−^ reducing agents.
— The direct effect of the chelation of Pro towards transition metal ions is evaluated through the reactions between the most stable complexes of Pro (i.e. Fe-Pro and Cu-Pro at **Site-2**) with HO^●^ radical. As a result, the FHT reactions of the high oxidation state Fe(III) and Cu(II) complexes at H10 and H12 sites occur at slower rate constants than those of the free Pro. Meanwhile, the rate constants of the SET reaction of these complexes are negligible. Conversely, the rate constants of the low oxidation state-based ion complexes (i.e. Fe(II) and Cu(I)) are determined to be higher than those of the free ligand.
— The HO^●^-initiated oxidation of Pro occurs via the hydrogen atom transfer (HAT) process. The complexation of free Pro towards Fe(III) and Cu(II) ions does not change the nature of their HAT processes.

Hopefully, the obtained results shed more light on the chemical mechanism of the L-proline amino acid oxidation by HO^●^ radical and the effects of the complexation with the transition metal ions on the oxidation process. They contribute to a better understanding of the origin of ageing and different diseases resulting from the accumulation of carbonyl derivatives caused by protein oxidation.

## Data Availability

All relevant necessary data to reproduce all results in the paper are within the main text, electronic supplementary material [69] and the Dryad Digital Repository: https://doi.org/10.5061/dryad.k6djh9wbb [70].

## References

[1] Areski F et al. 2019 Norine: update of the nonribosomal peptide resource. Nucleic Acids Res. **48**, D465-D469. (10.1093/nar/gkz1000) PMC7145658 31691799

[2] Bröcker MJ, Ho JML, Church GM, Söll D, O'Donoghue P. 2014 Recoding the Genetic Code with Selenocysteine. Angew. Chemie Int. Ed. **53**, 319-323. (10.1002/anie.201308584) PMC4004526 24511637

[3] Young VR. 1994 Adult amino acid requirements: the case for a major revision in current recommendations. J. Nutr. **124**, 1517S-1523S. (10.1093/jn/124.suppl_8.1517S) 8064412

[4] Hawkins AJS. 1991 Protein turnover: a functional appraisal. Funct. Ecol. **5**, 222. (10.2307/2389260)

[5] Kaya S, Tüzün B, Kaya C, Obot IB. 2016 Determination of corrosion inhibition effects of amino acids: quantum chemical and molecular dynamic simulation study. J. Taiwan Inst. Chem. Eng. **58**, 528-535. (10.1016/j.jtice.2015.06.009)

[6] Stadtman ER, Berlett BS. 1991 Fenton chemistry. Amino acid oxidation. J. Biol. Chem. **266**, 17 201-17 211. (10.1016/S0021-9258(19)47359-6) 1894614

[7] McGregor KG, Anastasio C. 2001 Chemistry of fog waters in California's Central Valley: 2. Photochemical transformations of amino acids and alkyl amines. Atmos. Environ. **35**, 1091-1104. (10.1016/S1352-2310(00)00282-X)

[8] Wen L, Schaefer T, Zhang Y, He L, Ventura ON, Herrmann H. 2022 T- and pH-dependent OH radical reaction kinetics with glycine, alanine, serine, and threonine in the aqueous phase. Phys. Chem. Chem. Phys. **24**, 11 054-11 065. (10.1039/D1CP05186E) 35471651

[9] Domingues MRM, Domingues P, Reis A, Fonseca C, Amado FML, Ferrer-Correia AJ. 2003 Identification of oxidation products and free radicals of tryptophan by mass spectrometry. J. Am. Soc. Mass Spectrom. **14**, 406-416. (10.1016/S1044-0305(03)00127-2) 12686488

[10] Ito T, Morimoto S, Fujita S, Nishimoto S. 2009 Radical intermediates generated in the reactions of l-arginine with hydroxyl radical and sulfate radical anion: a pulse radiolysis study. Radiat. Phys. Chem. **78**, 256-260. (10.1016/j.radphyschem.2009.01.005)

[11] Morimoto S, Ito T, Fujita S, Nishimoto S. 2008 A pulse radiolysis study on the reactions of hydroxyl radical and sulfate radical anion with guanidine derivatives in aqueous solution. Chem. Phys. Lett. **461**, 300-304. (10.1016/j.cplett.2008.07.013)

[12] Uranga J, Mujika JI, Matxain JM. 2015 ·OH oxidation toward S- and OH-containing amino acids. J. Phys. Chem. B **119**, 15 430-15 442. (10.1021/acs.jpcb.5b09825) 26588251

[13] Uranga J, Mujika JI, Grande-Aztatzi R, Matxain JM. 2018 Oxidation of acid, base, and amide side-chain amino acid derivatives via hydroxyl radical. J. Phys. Chem. B **122**, 4956-4971. (10.1021/acs.jpcb.7b12450) 29676577

[14] Scheiner S, Kar T. 2010 Analysis of the reactivities of protein C−H bonds to H atom abstraction by OH radical. J. Am. Chem. Soc. **132**, 16 450-16 459. (10.1021/ja105204v) 21047072

[15] Yamauchi O, Odani A, Takani M. 2002 Metal–amino acid chemistry. Weak interactions and related functions of side chain groups. J. Chem. Soc., Dalt. Trans. **18**, 3411-3421. (10.1039/B202385G)

[16] Elius Hossain M, Mahmudul Hasan M, Halim MEMA, Ehsan MQ, Halim MEMA. 2015 Interaction between transition metals and phenylalanine: a combined experimental and computational study. Spectrochim. Acta Part A Mol. Biomol. Spectrosc. **138**, 499-508. (10.1016/j.saa.2014.11.084) 25528509

[17] Niklas N, Hampel F, Alsfasser R. 2003 Dipicolylglycyl-phenylalanine zinc(ii): a metallopeptide with a built-in conformational switch and its homochiral helical coordination polymer. Chem. Commun. **13**, 1586. (10.1039/b303172a) 12868766

[18] Listyarini RV, Gesto DS, Paiva P, Ramos MJ, Fernandes PA. 2019 Benchmark of density functionals for the calculation of the redox potential of Fe3+/Fe2+ within protein coordination shells. Front. Chem. **7**, 1-12. (10.3389/fchem.2019.00391) 31231631 PMC6560050

[19] Binolfi A et al. 2010 Bioinorganic chemistry of Parkinson's Disease: structural determinants for the copper-mediated amyloid formation of alpha-synuclein. Inorg. Chem. **49**, 10 668-10 679. (10.1021/ic1016752) 20964419

[20] Abdel-Mottaleb MSA, Ismail EH. 2019 Transition metal complexes of mixed bioligands: synthesis, characterization, DFT modeling, and applications. J. Chem. **2019**, 1-18. (10.1155/2019/3241061)

[21] Milach OA, Mel'sitova IV, Yurkova IL. 2020 Pro(anti)oxidant Properties of Amino Acids and Their Derivatives in The Presence of Fe(II) and Cu(II) Ions. Russ. J. Gen. Chem. **90**, 987-993. (10.1134/S1070363220060080)

[22] Ka H, Yi B, Kim M-J, Lee J. 2016 Evaluation of antioxidant or prooxidant properties of selected amino acids using in vitro assays and in oil-in-water emulsions under riboflavin sensitization. J. Food Sci. **81**, C1118-C1123. (10.1111/1750-3841.13304) 27095610

[23] Truong DH, Ngo TC, Nguyen THL, Dao DQ. 2022 Oxidation of L-leucine amino acid initiated by hydroxyl radical: are transition metal ions an enhancement factor? R. Soc. Open Sci. **9**, 220316. (10.1098/rsos.220316) 36117865 PMC9470255

[24] Wu G et al. 2011 Proline and hydroxyproline metabolism: implications for animal and human nutrition. Amino Acids **40**, 1053-1063. (10.1007/s00726-010-0715-z) 20697752 PMC3773366

[25] Kaul S, Sharma SS, Mehta IK. 2008 Free radical scavenging potential of L-proline: evidence from in vitro assays. Amino Acids **34**, 315-320. (10.1007/s00726-006-0407-x) 17086481

[26] Hasanuzzaman M, Alam MM, Rahman A, Hasanuzzaman M, Nahar K, Fujita M. 2014 Exogenous proline and glycine betaine mediated upregulation of antioxidant defense and glyoxalase systems provides better protection against salt-induced oxidative stress in two rice (Oryza sativa L.) Varieties. Biomed Res. Int. **2014**, 1-17. (10.1155/2014/757219) PMC4065706 24991566

[27] Mehta SK, Gaur JP. 1999 Heavy-metal-induced proline accumulation and its role in ameliorating metal toxicity in Chlorella vulgaris. New Phytol. **143**, 253-259. (10.1046/j.1469-8137.1999.00447.x)

[28] Stadtman ER, Berlett BS. 1998 Reactive oxygen-mediated protein oxidation in aging and disease. Drug Metab. Rev. **30**, 225-243. (10.3109/03602539808996310) 9606602

[29] Stadtman ER. 2006 Protein oxidation and aging. Free Radic. Res. **40**, 1250-1258. (10.1080/10715760600918142) 17090414

[30] Chapman ML, Rubin BR, Gracy RW. 1989 Increased carbonyl content of proteins in synovial fluid from patients with rheumatoid arthritis. J. Rheumatol. **16**, 15-18. 2716005

[31] Murphy ME, Kehrer JP. 1989 Oxidation state of tissue thiol groups and content of protein carbonyl groups in chickens with inherited muscular dystrophy. Biochem. J. **260**, 359-364. (10.1042/bj2600359) 2764876 PMC1138677

[32] Carney JM, Smith CD, Carney AM, Butterfield DA. 1994 Aging- and oxygen-induced modifications in brain biochemistry and behavior. Ann. N. Y. Acad. Sci. **738**, 44-53. (10.1111/j.1749-6632.1994.tb21788.x) 7832454

[33] Salamone M, Basili F, Bietti M. 2015 Reactivity and selectivity patterns in hydrogen atom transfer from amino acid C-H bonds to the cumyloxyl radical: Polar effects as a rationale for the preferential reaction at proline residues. J. Org. Chem. **80**, 3643-3650. (10.1021/acs.joc.5b00549) 25774567

[34] Signorelli S, Coitiño EL, Borsani O, Monza J. 2014 Molecular mechanisms for the reaction between •OH radicals and proline: Insights on the role as reactive oxygen species scavenger in plant stress. J. Phys. Chem. B **118**, 37-47. (10.1021/jp407773u) 24328335

[35] Frisch MJ et al. 2016 Gaussion 16 Rev. A.03. Wallingford, CT: Gaussian Inc.

[36] Zhao Y, Schultz NE, Truhlar DG. 2006 Design of density functionals by combining the method of constraint satisfaction with parametrization for thermochemistry, thermochemical kinetics, and noncovalent interactions. J. Chem. Theory Comput. **2**, 364-382. (10.1021/ct0502763) 26626525

[37] Frisch MJ, Pople JA, Binkley JS. 1984 Self-consistent molecular orbital methods 25. Supplementary functions for Gaussian basis sets. J. Chem. Phys. **80**, 3265-3269. (10.1063/1.447079)

[38] Alecu IM, Zheng J, Zhao Y, Truhlar DG. 2010 Computational thermochemistry: scale factor databases and scale factors for vibrational frequencies obtained from electronic model chemistries. J. Chem. Theory Comput. **6**, 2872-2887. (10.1021/ct100326h) 26616087

[39] Malacaria L, Corrente GA, Beneduci A, Furia E, Marino T, Mazzone G. 2021 A Review on coordination properties of Al(III) and Fe(III) toward natural antioxidant molecules: experimental and theoretical insights. Molecules **26**, 2603. (10.3390/molecules26092603) 33946938 PMC8124610

[40] Mazzone G. 2019 On the inhibition of hydroxyl radical formation by hydroxycinnamic acids: the case of caffeic acid as a promising chelating ligand of a ferrous ion. J. Phys. Chem. A **123**, 9560-9566. (10.1021/acs.jpca.9b08384) 31603328

[41] Truong DH et al. 2022 New insights into the competition between antioxidant activities and pro-oxidant risks of rosmarinic acid. RSC Adv. **12**, 1499-1514. (10.1039/D1RA07599C) 35425185 PMC8978883

[42] Marenich AV, Cramer CJ, Truhlar DG. 2009 Universal solvation model based on solute electron density and on a continuum model of the solvent defined by the bulk dielectric constant and atomic surface tensions. J. Phys. Chem. B **113**, 6378-6396. (10.1021/jp810292n) 19366259

[43] Galano A, Tan DX, Reiter RJ. 2013 On the free radical scavenging activities of melatonin's metabolites, AFMK and AMK. J. Pineal Res. **54**, 245-257. (10.1111/jpi.12010) 22998574

[44] Galano A, Alvarez-Idaboy JR. 2013 A computational methodology for accurate predictions of rate constants in solution: application to the assessment of primary antioxidant activity. J. Comput. Chem. **34**, 2430-2445. (10.1002/jcc.23409) 23939817

[45] García-Díez G, Mora-Diez N. 2020 Theoretical study of the iron complexes with aminoguanidine: investigating secondary antioxidant activity. Antioxidants **9**, 756. (10.3390/antiox9080756) 32824195 PMC7463863

[46] Truong DH, Nhung NTA, Dao DQ. 2020 Iron ions chelation-based antioxidant potential vs. pro-oxidant risk of ferulic acid: a DFT study in aqueous phase. Comput. Theor. Chem. **1185**, 112905. (10.1016/j.comptc.2020.112905)

[47] Ngo TC, Truong DH, Nguyen TTN, Quang DT, Dao DQ. 2022 On the free radical scavenging and metallic ion chelating activities of pyridoxal - Could the pro-oxidant risk be competitive? Phytochemistry **199**, 113176. (10.1016/j.phytochem.2022.113176) 35390394

[48] Mora-Diez N, Monreal-Corona R, Biddlecombe J, Ippolito A. 2020 Theoretical study of the iron complexes with lipoic and dihydrolipoic acids: exploring secondary antioxidant activity. Antioxidants **9**, 1-21. (10.3390/antiox9080674) PMC7465238 32731543

[49] García-Díez G, Monreal-Corona R, Mora-Diez N. 2021 Complexes of copper and iron with pyridoxamine, ascorbic acid, and a model amadori compound: exploring pyridoxamine's secondary antioxidant activity. Antioxidants **10**, 1-20. (10.3390/antiox10020208) PMC7912584 33535448

[50] El Amrani FBA, Perelló L, Real JA, González-Alvarez M, Alzuet G, Borrás J, García-Granda S, Montejo-Bernardo J. 2006 Oxidative DNA cleavage induced by an iron(III) flavonoid complex: synthesis, crystal structure and characterization of chlorobis(flavonolato)(methanol) iron(III) complex. J. Inorg. Biochem. **100**, 1208-1218. (10.1016/j.jinorgbio.2006.01.036) 16527356

[51] Furia E, Beneduci A, Russo N, Marino T. 2018 Structural characterization of aluminium(III) and iron(III) complexes of coumarinic acid in aqueous solutions from combined experimental and theoretical investigations. New J. Chem. **42**, 11 006-11 012. (10.1039/c8nj01244j)

[52] Pamidighantam S, Nakandala S, Abeysinghe E, Wimalasena C, Yodage SR, Marru S, Pierce M. 2016 Community science exemplars in SEAGrid Science gateway: apache airavata based implementation of advanced infrastructure. Procedia Comput. Sci. **80**, 1927-1939. (10.1016/j.procs.2016.05.535)

[53] Shen N, Fan Y, Pamidighantam S. 2014 E-science infrastructures for molecular modeling and parametrization. J. Comput. Sci. **5**, 576-589. (10.1016/j.jocs.2014.01.005)

[54] Dooley R, Milfeld K, Guiang C, Pamidighantam S, Allen G. 2006 From proposal to production: lessons learned developing the computational chemistry grid cyberinfrastructure. J. Grid Comput. **4**, 195-208. (10.1007/s10723-006-9043-7)

[55] Milfeld K, Guiang C, Pamidighantam S, Giuliani J. 2005 Cluster computing through an application-oriented Computational Chemistry Grid. In Proc. 2005 Linux Clust. HPC Revolut.

[56] Galano A, Alvarez-Idaboy JR, Bravo-Pérez G, Ruiz-Santoyo ME. 2002 Mechanism and rate coefficients of the gas phase OH hydrogen abstraction reaction from asparagine: a quantum mechanical approach. J. Mol. Struct. Theochem **617**, 77-86. (10.1016/S0166-1280(02)00388-3)

[57] Galano A, Alvarez-Idaboy JR, Cruz-Torres A, Ruiz-Santoyo ME. 2003 Kinetics and mechanism of the gas-phase OH hydrogen abstraction reaction from methionine: a quantum mechanical approach. Int. J. Chem. Kinet. **35**, 212-221. (10.1002/kin.10117)

[58] Galano A, Alvarez-Idaboy JR, Cruz-Torres A, Ruiz-Santoyo ME. 2003 Rate coefficients and mechanism of the gas phase OH hydrogen abstraction reaction from serine: a quantum mechanical approach. J. Mol. Struct. Theochem **629**, 165-174. (10.1016/S0166-1280(03)00140-4)

[59] Medina ME, Galano A, Alvarez-Idaboy JR. 2015 Site reactivity in the free radicals induced damage to leucine residues: a theoretical study. Phys. Chem. Chem. Phys. **17**, 4970-4976. (10.1039/C4CP05688D) 25592549

[60] Liochev SI, Fridovich I. 2002 The Haber-Weiss cycle - 70 years later: an alternative view. Redox Rep. **7**, 55-57. (10.1179/135100002125000190) 11981456

[61] Waltz WL, Akhtar SS, Eager RL. 1973 Oxidation of some transition-metal cyanide compounds by hydroxyl radical. Can. J. Chem. **51**, 2525-2529. (10.1139/v73-380)

[62] Zehavi D, Rabani J. 1972 Pulse radiolysis of the aqueous ferro-ferricyanide system. 1. Reactions of OH, HO2, and O2- radicals. J. Phys. Chem. **76**, 3703-3709. (10.1021/j100669a006)

[63] Galano A. 2017 Free radicals induced oxidative stress at a molecular level: the current status, challenges and perspectives of computational chemistry based protocols. J. Mex. Chem. Soc. **59**, 231-262. (10.29356/jmcs.v59i4.81)

[64] Sirjoosingh A, Hammes-Schiffer S. 2011 Proton-coupled electron transfer versus hydrogen atom transfer: generation of charge-localized diabatic states. J. Phys. Chem. A **115**, 2367-2377. (10.1021/jp111210c) 21351757

[65] Carreon-Gonzalez M, Muñoz-Rugeles L, Vivier-Bunge A, Alvarez-Idaboy JR. 2022 Chemical repair of damaged leucine and tryptophane by thiophenols at close to diffusion-controlled rates: mechanisms and kinetics. J. Comput. Chem. **43**, 556-567. (10.1002/jcc.26813) 35106786

[66] Hammes-Schiffer S et al. 2015 Proton-coupled electron transfer guidelines, fair and square. J. Am. Chem. Soc. **110**, 16 655-16 663. (10.1039/c2sc20115a)

[67] Skone JH, Soudackov AV, Hammes-Schiffer S. 2006 Calculation of vibronic couplings for phenoxyl/phenol and benzyl/toluene self-exchange reactions: implications for proton-coupled electron transfer mechanisms. J. Am. Chem. Soc. **128**, 16 655-16 663. (10.1021/ja0656548) 17177415

[68] Huynh MHV, Meyer TJ. 2007 Proton-coupled electron transfer. Chem. Rev. **107**, 5004-5064. (10.1021/cr0500030) 17999556 PMC3449329

[69] Truong DH, Lan Nguyen TH, Dao DQ. 2023 Revisiting the HO^●^-initiated oxidation of L-proline amino acid in the aqueous phase: influence of transition metal ions. *Figshare*. (10.6084/m9.figshare.c.6673588) PMC10245202 37293362

[70] Truong DH, Lan Nguyen TH, Dao DQ. 2023 Data from: Revisiting the HO^●^-initiated oxidation of L-proline amino acid in the aqueous phase: influence of transition metal ions. Dryad Digital Repository. (10.5061/dryad.k6djh9wbb) PMC10245202 37293362

